# A comparison of noninvasive and invasive acupuncture in preventing postoperative nausea and vomiting: A protocol for systematic review and Bayesian network meta-analysis

**DOI:** 10.1097/MD.0000000000021544

**Published:** 2020-07-31

**Authors:** Cheng-Wei Fu, Qing Shu, Yang Jiao, Tong Wu, Ai-Qun Song, Qiao-Chu Zhu, Wei-Ping Zhang

**Affiliations:** a Zhuji People's Hospital, Zhuji; b Zhongnan Hospital of Wuhan University; c Hubei Provincial Hospital of Traditional Chinese Medicine; d Hubei Province Academy of Traditional Chinese Medicine; e Hubei University of Traditional Chinese Medicine, Wuhan, China.

**Keywords:** acupuncture therapy, network meta-analysis, postoperative nausea and vomiting

## Abstract

**Background:**

Postoperative nausea and vomiting (PONV) is a main complication of surgery, and by now, drugs cannot prevent it completely. Some meta-analyses have proved acupuncture therapy can prevent PONV. However, it is still controversial whether noninvasive acupuncture therapies are comparable with invasive ones. This study uses Bayesian network meta-analysis to compare the effectiveness of different forms of acupuncture in preventing PONV.

**Methods:**

PubMed/Medline, Cochrane library, Web of Science, Ebsco, Ovid/Embase, China National Knowledge Infrastructure, Wanfang Database, VIP Database, and China Biology Medicine disc will be searched from inception to May 2020. All randomized control trails meet the criterion will be included. Quality evaluation of included studies will be implemented with Cochrane risk-of-bias tool. STATA 14.0 will be used to perform pairwise meta-analysis. Addis 1.16.8, R 3.6.3, OpenBUGS 3.2.3, and STATA 14.0 will be used to conduct network meta-analysis. The evidence will be assessed by the grading of recommendations assessment, development, and evaluation approach using GRADE Profiler 3.6.

**Results:**

The results of this review will be submitted to a peer-reviewed journal for publication and generate a comprehensive review of current evidence.

**Conclusion:**

Our results will help to improve the clinical decision-making ability and policy-making in PONV domain.

**Systematic review registration:**

The protocol has been registered on INPLASY 202060108.

## Abstract 2

Supplemental Digital Content is available in the text

## 1 Introduction

Nowadays, countless surgical procedures are carried out annually with the help of anesthesia worldwide.^[1]^ Among all complications, postoperative nausea and vomiting (PONV) and postoperative pain account for over half of reported symptoms by surgical patients.^[2]^ Apfel et al^[3]^ gives 4 main risk factors of PONV which help anesthetists recognize those patients under low or high risk. It is reported that the possibility of PONV can raise to 80% in high-risk patients.^[4]^ PONV is not fatal, but when dehydration, electrolyte imbalance, and esophageal rupture come across, the situation will nosedive and even cause death.^[5]^ It is reported that predicting scoring systems may only have poor to moderate accuracy.^[6]^ And a recent meta-analysis shows only postoperative opioids will increase PONV rather than preoperative and intraoperative.^[7]^ Due to the low capacity of prediction, clinicians have to treat patients as many as possible with PONV's prophylactic drugs. Taking all antiemetics into consideration, 5-hydroxytryptamine receptor antagonists (5HTRA) is recommended as the first-chosen antiemetics by FDA.^[8]^ Su et al summarized common antiemetics for PONV and indicated that still a third patients will suffer from headache, liver enzymes, constipation, and even QT interval by taking 5HTRA.^[6,9]^ Though it is a consensus that moderate-to-high risk patients should receive prophylaxis with combination therapy or a multimodal approach, it still remains inclusive to establish a perfect protocol for preventing PONV.^[8]^ And thinking in patients’ position, in order to avoid PONV, they have no better choice but to pay an extra 30 to 200 dollars which is an enormous financial burden.^[10,11]^

Thus, researchers shift their focus to complementary and alternative therapies, and find some of them^[12–14]^ can help reduce the incidence rate of PONV. Among all regimens, acupuncture is regarded as a promising non-pharmacological technique, and stimulation of Neiguan (PC6) shows confident potential in alleviating PONV supported by some evidence-based studies.^[1,15]^ However, the adverse effects like bleeding, discomfort, and residual pain may hamper the successful development of traditional acupuncture therapy.^[16]^ and otherwise, modern acupuncture therapy such as transcutaneous electric nerve stimulation and acupressure, without piercing skin and feeling pain, is more and more popular and acceptable worldwide.^[17]^ Thus, we put forward that whether noninvasive regimens are comparison with invasive ones. If so, why we do not use a more considered receptive and safety method?

In this study, we will evaluate the effectiveness of invasive and noninvasive acupuncture therapies as many as possible using network meta-analysis (NMA) based on a Bayesian model and hope this work could inspire relevant study.

## 2 Methods

The protocol has been registered on INPLASY (https://inplasy.com/) and is waiting for a registered ID. We used the preferred reporting items for systematic review and meta-analysis protocols statement.^[18,19]^ Because this is a systematic literature research, ethical approval can be skipped.

### 2.1 Eligibility criteria

#### 2.1.1 Type of study

Only peer-reviewed randomized control trails will be eligible for inclusion. And language will be restricted to English and Chinese. Review, case report, protocol, animal study, supplementary issue, conference paper will be excluded.

#### 2.1.2 Participants

Adult patients undergoing surgery within general anesthesia will be considered. But those who used regional anesthesia (eg, superficial mass) or sedation as anesthetic techniques (eg, endoscopy) will not be included.

#### 2.1.3 Interventions

**Figure 1:**
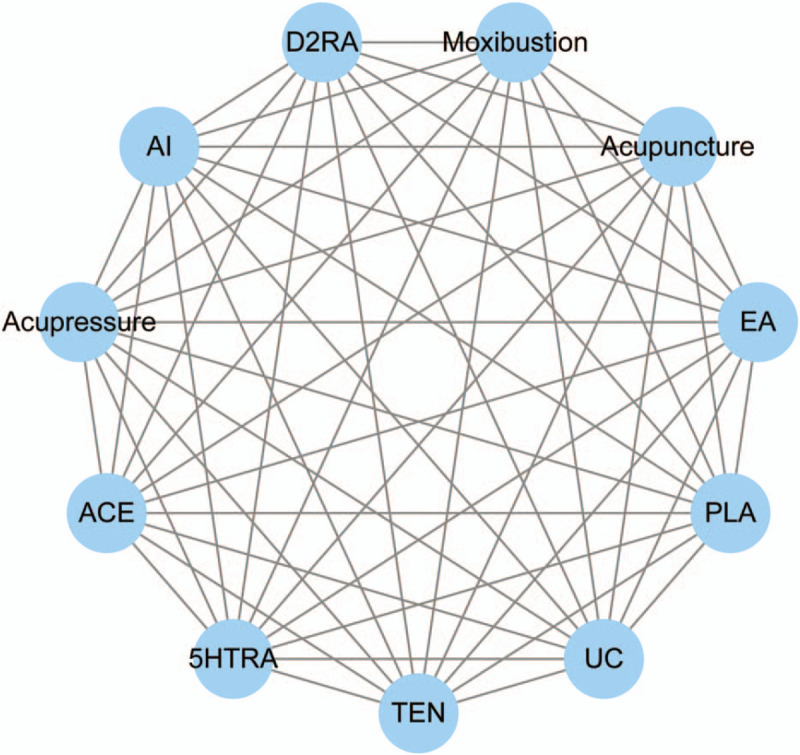
Network plot of possible direct comparisons. 5HTRA = 5-hydroxytryptamine receptor antagonist, ACE = acupoint catgut embedding, AI = acupoint injection, D2RA = dopamine D2 receptor antagonist, EA = electro-acupuncture, PLA = placebo, TEN = transcutaneous electrical stimulation, UC = usual care.

Any acupuncture therapy will be included, for instance, acupuncture, electro-acupuncture and moxibustion, and so on. In particular, pre-search showed that transcutaneous electric nerve stimulation and acupressure with acupoint are common in relevant studies, so they are also regarded as acupuncture therapies. Acupuncture therapy combined with antiemetics will also be recorded. And we defined invasive procedure as piercing the skin. Studies will be excluded that non-prophylactic use of acupuncture therapies or patients had been diagnosed as PONV before intervention. In addition, ear acupuncture will not be included for whose rationale is not on the bases of traditional Chinese medicine. Figure 1 gives example to illustrate a potential network plot.

#### 2.1.4 Control group

Control group consisted of usual care (means no treatment), sham acupuncture therapy, medication such (eg, 5HTRA). But other complementary or alternative therapy will be excluded (eg, ginger or aromatherapy).

#### 2.1.5 Outcomes

##### 2.1.5.1 Primary outcomes

The effectiveness will be recorded and primary endpoints are the incidences of postoperative nausea (PON), postoperative vomiting (POV), PONVs, and postoperative rescue antiemetics within 24 hours after surgery.

##### 2.1.5.2 Secondary outcomes

Other common endpoints will also be recorded, for instance, 6 hours, 12 hours, 48 hours. However, when less than 5 studies describe the same endpoints, we will not use meta-analysis.

### 2.2 Search strategies

Authors will search PubMed/Medline, Cochrane library, Web of Science, Ebsco, Ovid/Embase, China National Knowledge Infrastructure, Wanfang Database, VIP Database, and China Biology Medicine disc from setup time to April 2020. The search strategy will contain both PONV and acupuncture therapies including “acupuncture,” “electroacupuncture,” “acupuncture therapy,” “PONV,” “postoperative nausea and vomiting,” “PON,” “POV,” and similar terms. Search strategy will be adjusted according to various databases. Supplemental Digital Content (Appendix 1) gives a detailed search strategy of PubMed/Medline.

### 2.3 Study selection

**Figure 2:**
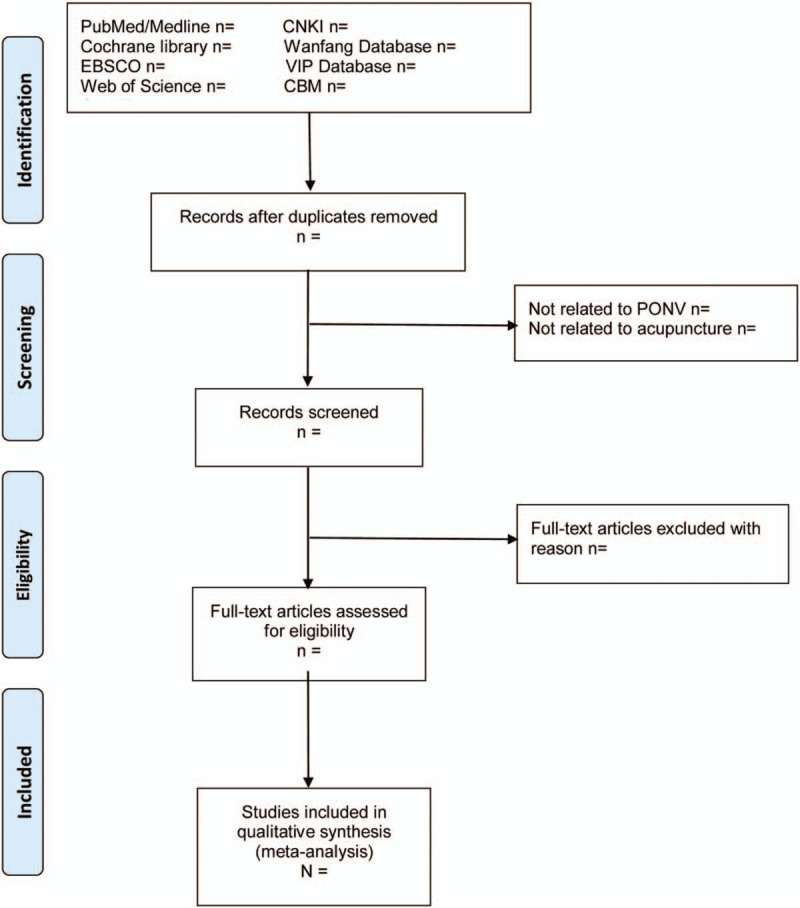
PRISMA flow diagram of the study selection process. PRISMA = preferred reporting items for systematic review and meta-analysis.

In order to ensure high inter-rater reliability, a predefined inclusion and exclusion criteria will be used. Two reviewers (Tong Wu, Qiao-Chu Zhu) will scan all studies independently according to Supplemental Digital Content (Appendix 2) and a third reviewer (Yang Jiao) will request adjudications if necessary. Only the most informative and complete study of any duplicate publications will be selected. The process of screening will be shown by preferred reporting items for systematic review and meta-analysis flow chart as Figure 2.

### 2.4 Data extraction

After identification of the target randomized control trails, 1 reviewer (Cheng-Wei Fu) will extract the following data into a database created by Excel 2019 and checked by the second reviewer (Qing Shu):

(1) studies information: title, first author, publication year, first author's country, ethical approval, and registration of clinical trial registry;
(2) patient information: sample size, sex, diseases, surgical spot, postoperative analgesia, American Society of Anesthesiologists Class, type of anesthesia, types of intervention, time, and acupoints;
(3) outcomes information: the incidences of PON, POV, PONVs, or POR within proper time horizon.

The third reviewer (Ai-Qun Song) is the referee in case of doubts or disagreements. In addition, if data are presented as figures, GetData Graph Digitizer will help us to extract the number.

### 2.5 Risk of bias assessment

Cochrane risk-of-bias tool (ROB 2.0) will be used to evaluate the quality.^[20]^ ROB 2.0 has 5 domains including:

(1) bias arising from the randomization process,
(2) bias due to deviations from intended interventions,
(3) bias due to missing outcome data,
(4) bias in measurement of the outcome,
(5) bias in selection of the reported result.

Finally, an overall risk of bias will be given based on above bias. Two reviewers (Cheng-Wei Fu, Qing Shu) will use ROB 2.0 to assess all matched studies and the third reviewer (Wei-Ping Zhang) will request adjudications if necessary.

### 2.6 Statistical analysis

#### 2.6.1 Pairwise meta-analysis

Only 3 or more studies comparing same interventions directly will be conducted in pairwise meta-analysis. Stata 14.0 will be used to solve pairwise meta-analysis, odds ratio, and 95% confidence interval will be adopted. Heterogeneity is quantified with the *I*^2^ statistic. When *I*^2^ > 50%, a random effect model will be adopted; if not, a fixed effect model. And before selecting model, sensitivity analysis will be accomplished if sufficient studies are available. When pairwise comparison studies ≥10, a Begg testing will be performed to explore the publication bias.

#### 2.6.2 NMA

NMA will be performed by Addis1.16.8, OpenBUGS 3.2.3, R 3.6.3 and STATA 14.0. OpenBUGS is based on Bayesian framework using the Markov Chain Monte Carlo theory.^[21]^ As the incidence rate is dichotomous data, odds ratio and 95% confidence interval will be adopted. Considering the extreme case report that response may be 0, we will add 0.5 to event rate artificially. Clinical heterogeneity will be assessed according to participants’ characteristics, interventions, and outcomes of the included trials. R will be used to assess the methodological heterogeneity. It gives *I*^2^ to evaluate the pooled network heterogeneity. Literatures that affect heterogeneity will be deleted if it appears a high heterogeneity. Convergence will be evaluated by potential scale reduction factor according to the Brooks–Gelman–Rubin method. We assessed global inconsistency by fitting both inconsistency model and consistency model. Then, node spilt analysis will be performed to assess local inconsistency by comparing direct and indirect effect. In addition, if loop inconsistency appears, it will be performed for a better demonstration of results. Subgroup analysis and regression will be finished if necessary. League figures will be used to demonstrate the results of multiple treatment comparisons. The surface under the cumulative ranking curve values will be used to rank the probabilities ranged from 0% to 100% (in this review, a lower rank is worse). Network funnel plot will be conducted to assess the publication bias.

#### 2.6.3 Quality of evidence

Quality of evidence will be evaluated by the grades of recommendations assessment development, and evaluation (GRADE) guidelines. There are 3 factors (residual confounding, dose-response gradient and large magnitude of effect) to promote the quality and 5 factors (study limitations, inconsistency, indirectness, publication bias and imprecision) to lower it and the quality will be graded in very low, low, moderate and high. GRADE profiler 3.6 will be used to conducted the assessment.

## 3 Conclusion

PONV management is still one of the main concerns in postoperative period which may lead to delays in the discharge and increase health expenditure. So far, none of existing drugs can prevent PONV completely^[22]^ while only 28% of patients can benefit from prophylactic use of antiemetics.^[23]^ Some low to moderate evidences have shown the effectiveness and safety of acupuncture therapy for the prevention of PONV,^[24,25]^ but it lacks studies which compare different acupuncture therapies, so that clinicians cannot judge the therapeutic value of different forms of regimens, which is not conducive to choose the best acupuncture treatment. In addition, it is controversial that whether noninvasive acupuncture therapies can be comparable with invasive ones. Our research is aimed at providing a clinically useful ranking of acupuncture interventions for PONV prophylaxis, as well as to provide credible evidence for initiative research directions. However, literatures written by languages other than Chinese and English will be eliminated, which will lead to some biases. Besides, the manipulation of time and the intensity of stimulation can exert influences on the effect of acupuncture therapy, we may reduce the inconsistency by setting subgroups or conducting meta-regression as necessary. Research results will be published in relevant journal and they may appeal to a broad audience, including anesthetists, surgeons, practice guideline developers, researchers, and policymakers. We will update this protocol required in the future and the date of amendments and description of changes will be presented as a supplement.

## Author contributions

**Conceptualization:** Wei-Ping Zhang, Cheng-Wei Fu, Yang Jiao.

**Data curation:** Wei-Ping Zhang, Cheng-Wei Fu, Yang Jiao.

**Formal analysis:** Cheng-Wei Fu.

**Funding acquisition:** Qing Shu.

**Investigation:** Tong Wu, Qiao-Chu Zhu.

**Methodology:** Wei-Ping Zhang, Cheng-Wei Fu, Qing Shu.

**Project administration:** Wei-Ping Zhang.

**Resources:** Wei-Ping Zhang, Cheng-Wei Fu.

**Software:** Wei-Ping Zhang, Cheng-Wei Fu.

**Supervision:** Yang Jiao, Ai-Qun Song.

**Validation:** Wei-Ping Zhang.

**Visualization:** Cheng-Wei Fu.

**Writing – original draft:** Wei-Ping Zhang, Cheng-Wei Fu.

**Writing – review & editing:** Cheng-Wei Fu, Tong Wu.

## Supplementary Material

Supplemental Digital Content

## Supplementary Material

Supplemental Digital Content
