# Supplementary material for: A comparison of noninvasive and invasive acupuncture in preventing postoperative nausea and vomiting: A protocol for systematic review and Bayesian network meta-analysis
Source: Medicine (Baltimore). 2020 Jul 31;99(31):e21544. doi: 10.1097/MD.0000000000021544 (PMC7402802; doi:10.1097/MD.0000000000021544)
Supplement: Supplemental Digital Content [file medi-99-e21544-s001.docx]

| Search Strategy of PubMed/Medline | |
| --- | --- |
| #1 | acupuncture[MeSH Terms] |
| #2 | moxibustion[MeSH Terms] |
| #3 | electroacupuncture[MeSH Terms] |
| #4 | acupuncture therapy[MeSH Terms] |
| #5 | acupressure[MeSH Terms] |
| #6 | transcutaneous electric nerve stimulation[MeSH Terms] |
| #7 | acupuncture[Title/Abstract] |
| #8 | moxibustion[Title/Abstract] |
| #9 | electroacupuncture[Title/Abstract] |
| #10 | acupuncture therapy[Title/Abstract] |
| #11 | acupressure[Title/Abstract] |
| #12 | transcutaneous electric nerve stimulation[Title/Abstract] |
| #13 | transcutaneous electric[Title/Abstract] |
| #14 | TEN[Title/Abstract] |
| #15 | TEA[Title/Abstract] |
| #16 | #1 OR #2 OR #3 OR #4 OR #5 OR #6 |
| #17 | #7 OR #8 OR#9 OR #10 OR #11 OR #12 OR#13 OR #14 OR #15 |
| #18 | #16 OR # 17 |
| #19 | nausea[Title/Abstract] |
| #20 | vomiting[Title/Abstract] |
| #21 | postoperative nausea[Title/Abstract] |
| #22 | postoperative vomiting[Title/Abstract] |
| #23 | retching[Title/Abstract] |
| #24 | vomit[Title/Abstract] |
| #25 | emesis[Title/Abstract] |
| #26 | PONV[Title/Abstract] |
| #27 | PON[Title/Abstract] |
| #28 | POV[Title/Abstract] |
| #29 | postoperative nausea and vomiting[Title/Abstract] |
| #30 | vomiting[MeSH Terms] |
| #31 | nausea[MeSH Terms] |
| #32 | postoperative nausea and vomiting[MeSH Terms] |
| #33 | #19 OR #20 OR #21 OR #22 OR #23 OR #24 OR #25 OR #26 OR #27 OR #28 OR #29 |
| #34 | #30 OR #31 OR #32 |
| #35 | #33 OR #34 |
| #36 | #18 AND #35 |

**Appendix 1 Draft Search Strategy**
