# Supplementary material for: A comparison of noninvasive and invasive acupuncture in preventing postoperative nausea and vomiting: A protocol for systematic review and Bayesian network meta-analysis
Source: Medicine (Baltimore). 2020 Jul 31;99(31):e21544. doi: 10.1097/MD.0000000000021544 (PMC7402802; doi:10.1097/MD.0000000000021544)
Supplement: Supplemental Digital Content [file medi-99-e21544-s002.docx]

**Appendix 2: Draft Eligibility Criteria**

Primary screening

1. Does this study is a clinical study?

YES____ NO____ UNCLEAR____

2. Does this study is a randomized control trail?

YES____ NO____ UNCLEAR____

3. Does this study describes patients undergo surgery?

YES____ NO____ UNCLEAR____

4. Does this study use acupuncture therapy?

YES____ NO____ UNCLEAR____

5. Does this study pays attention to postoperative nausea and vomiting?

YES____ NO____ UNCLEAR____

6. Does this study is written in Chinese or English?

YES____ NO____ UNCLEAR____

If you answer NO to any of these quesitions, the citation/study will be excluded. All other citations will be included.Secondary Screening

1. Does this study include adult patients (aged ≥ 18 years) undergo surgery?

YES____ NO____ UNCLEAR____

2. Does this study provides a specific general anesthesia protocol?

YES____ NO____ UNCLEAR____

3. Does acupuncture therapy is used as prophylaxis in this study?

YES____ NO____ UNCLEAR____

4. Does this study use only acupuncture therapy or combines antiemetics?

YES____ NO____ UNCLEAR____

5. Does this study records postoperative nausea and vomiting as result?

YES____ NO____ UNCLEAR____

6. Does this study is a randomized control trail?

YES____ NO____ UNCLEAR____

7. Does this study use only acupuncture therapy or combines antiemetics?

YES____ NO____ UNCLEAR____

8. Does this study has available data?

YES____ NO____ UNCLEAR____

If you answer NO to any of these questions, the citation/study will be excluded. All other full-text articles will be included.
